# Is individual day-to-day variation of arterial stiffness associated with variation of maximal aerobic performance?

**DOI:** 10.1186/s13102-021-00231-1

**Published:** 2021-01-09

**Authors:** Takanobu Okamoto, Ryota Kobayashi, Yuto Hashimoto, Naoki Kikuchi, Shigehiko Ogoh

**Affiliations:** 1 Department of Exercise Physiology, Nippon Sport Science University, 7-1-1, Fukasawa, Setagaya-ku, Tokyo, 158-8508 Japan; 2 Center for Fundamental Education, Teikyo University of Science, Tokyo, Japan; 3 Department of Training Science, Nippon Sport Science University, Tokyo, Japan; 4 Department of Biomedical Engineering, Toyo University, Saitama, Japan

**Keywords:** Pulse wave velocity, Maximal oxygen uptake, 1500-m time trial, Heart rate, Blood pressure

## Abstract

**Background:**

Maximal aerobic capacity, e.g. maximal oxygen uptake (V̇O_2_max), is not constant, and it has a time-dependent variation based on the condition of individual. On the other hand, arterial properties play an important role in determining aerobic performance, and lower arterial stiffness is associated with higher cardiorespiratory fitness levels. This study examined whether individual variations in maximal aerobic performance are associated with arterial stiffness.

**Methods:**

Twenty-four (mean age, 19.8 ± 0.2 y) and 10 (mean age, 21.2 ± 0.2 y) recreationally active young men and women participated in Experiment 1 (Ex1) and in Experiment 2 (Ex2), respectively. Aerobic performance was assessed using a graded power test (Ex1) or a 1500-m time trial (Ex2). Simultaneously, brachial-ankle pulse wave velocity (baPWV) was measured as an index of arterial stiffness in both Ex1 and Ex2 before the exercise trials. In both experiments, subjects returned for measurement of baPWV and V̇O_2_max or 1500-m time trial at 1 month after first measurements.

**Results:**

No significant differences in mean baPWV, V̇O_2_max or 1500-m run time were seen between first and second visits. Mean baPWV was significantly lower on days when participants showed higher V̇O_2_max or better 1500-m run time (*P* = 0.001 each) than on days when participants showed lower V̇O_2_max or worse 1500-m run time. In addition, a significant relationship was seen between individual changes in baPWV from first to second visits and changes in V̇O_2_max (*P*=0.0001) or 1500-m run time (*P*=0.04).

**Conclusion:**

These findings suggest that individual day-to-day variations in maximal aerobic performance are associated with variations in arterial stiffness.

**Supplementary Information:**

The online version contains supplementary material available at 10.1186/s13102-021-00231-1.

## Background

One of the main physiological determinants of maximal aerobic exercise performance is maximal oxygen uptake (V̇O_2_max), as the greatest volume of oxygen that an individual can take up and use during intense exercise [1]. V̇O_2_max is a key determinant of exercise performance during intense activity for an extended period of time and is frequently measured as an indicator of individual cardiorespiratory fitness [2–5].

Many previous studies have reported day-to-day variation in V̇O_2_max [6–10]. For example, it has been reported that day-to-day variation in V̇O_2_max determined by a graded power test using a cycle ergometer [10] or treadmill [6] was around 6% in healthy young adults, with no sex difference. This kind of day-to-day individual variation is an important reliability measure when sports professionals and/or scientists monitor exercise performance [11].

Some previous studies have suggested that arterial properties play an important role in determining aerobic performance, and that lower arterial stiffness is associated with higher cardiorespiratory fitness levels [12–16], although this is not a universal findings [17]. In addition, Roos et al. [18] reported that lower arterial stiffness contributed to increased V̇O_2_ through increases in myocardial flow reserve and myocardial perfusion, implying an association between higher cardiorespiratory fitness and lower arterial stiffness. Moreover, increased arterial stiffness induces a decrease in arterial function that buffers the pulsatile flow generated by the heart with each contraction and does not ensure continuous tissue perfusion in peripheral blood vessels, which may ultimately limit O_2_ delivery to working muscles, resulting in decreased exercise performance [19]. In addition, it has been reported that there is day-to-day variability of pulse wave velocity (PWV), which is a common method for measuring arterial stiffness [20–22]. Thus, changes in arterial stiffness may be one determinant of aerobic performance.

The aim of the present study was to examine whether individual variations in maximal aerobic performance are associated with variations in arterial stiffness. We hypothesized that even individual day-to-day variation in maximal aerobic performance is associated with variation of arterial stiffness. To test this hypothesis, the present study measured brachial-ankle PWV (baPWV) as an index of systemic arterial stiffness, along with V̇O_2_max or 1500-m run time. Measurements were made twice, on different days with an interval of 1 month, and the relationships between day-to-day individual variations in baPWV and variations in V̇O_2_max or 1500-m run time were assessed.

## Methods

### Participants

The present study consisted of two studies: Experiment 1 (Ex1) and Experiment 2 (Ex2). We used G*power software (version 3.1.5.1; Heinrich-Heine University, Dusseldorf, Germany) to calculate a sample size necessary to yield an estimated effect size of 0.8, an alpha level of 0.05, and statistical power of 0.8 for a paired t-test between the two trials [23]. This estimation revealed 15 participants as the number necessary for appropriate evaluation. Twenty-four recreationally active adults (20 males, 4 females; age, 19.8 ± 1. 2 y; range, 18–22 y; mean height, 167.9 ± 7.5 cm; mean weight, 68.6 ± 8.4 kg; mean body fat, 16.4 ± 6.1%, mean ±standard deviation [SD]) participated in Ex1, which involved a graded power test on a cycle ergometer as well as PWV measurement. Separately, 10 different recreationally active adults (7 males, 3 females; mean age, 21.2 ± 0.6 y; range, 18–22 y; mean height, 168.2 ± 3.7 cm; mean weight, 61.9 ± 4.5 kg; mean body fat, 14.9% ± 4.6%) participated in Ex2, which involved a 1500-m run as well as PWV measurement. All participants had performed recreationally activity (e.g., walking, jogging, cycling, etc.) in 1–2 sessions per week prior to starting the study. The International Physical Activity Questionnaire-Short Form was used to assess physical activity. In both experiments, all participants showed normal BP (< 130/85 mmHg). Exclusion criteria were: smoking; obesity (body mass index > 30 kg/m^2^); diseases or disorders affecting physical activity; pharmacotherapy compromising the cardiovascular system, including antihyperlipidemic, antihypertensive, or antihyperglycemic medications; pregnancy in females; or administration of oral contraceptives. No participants reported a history of cardiovascular disease.

Written, informed consent was obtained from all participants after receiving a full explanation of the study purpose and experimental procedures. The present study was conducted in accordance with the Declaration of Helsinki and its later amendments, and with the guidelines for experimental studies involving human participants published by our institutional review board (014-H34).

### Study design

This study examined the effects of arterial stiffness on maximal aerobic capacity. We tested the hypothesis that short-term individual variation in arterial stiffness affects cardiorespiratory fitness capacity and aerobic performance. In both experiments, participants visited the laboratory twice to perform the experimental protocol. Both visits were separated by about 1-month intervals (mean, 28 days). All female participants participated in the experiments during the early follicular phase of their menstrual cycle at about 1-month intervals to minimize any potential hormonal effects. To unify the measurement period, a measurement interval of 1 month was also applied to male participants. To eliminate any potential effects of food intake, all measurements were recorded at the same time (08:00–11:00) after overnight fasting. All participants were instructed to abstain from engaging in intense physical activity or consuming alcohol within 48 h of the beginning of the study, and from consuming any caffeine-containing beverages within 24 h of the beginning of the study. The participants were also instructed to maintain their regular lifestyle and not to change their diet, engage in any activities such as massages or stretching, or take any pharmacotherapies during the experimental period. To ensure the same level of physical effort during measurements, participants remained blinded to the results until completion of the second measurement.

In consideration of the effects of diurnal variation, measurements of baPWV, brachial BP, and heart rate (HR) were taken in a quiet room at a constant temperature (23–25 °C) at around the same time. By contrast, baPWV, brachial BP, and HR measurements were obtained before each exercise protocol. After the measurements, the participants performed an aerobic performance test to assess maximal aerobic power using a graded power test on a cycle ergometer (Ex1) or a 1500-m run (Ex2). The same investigator measured all parameters. We defined the change in V̇O_2_max or 1500-m run time between first and second visits as its variability in this study.

### Measurement of V̇O_2_max

We conducted a graded power test using an electronically braked cycle ergometer (Corival 1000ss; Lode Co., Groningen, the Netherlands) as an aerobic performance test to assess V̇O_2_max. We started the test at 30 W and then increased the workload by 15 W every minute until exhaustion. V̇O_2_max, carbon dioxide production (V̇CO_2_), and respiratory exchange ratio (RER: measured as V̇CO_2_/V̇O_2_) was monitored breath-by-breath using a metabolic measurement cart (AE-310S; Minato Medical Science, Osaka, Japan). V̇O_2_max was defined as the highest 30-s averaged oxygen consumption when VO_2_ plateaued, concurrent with a RER > 1.15 [24].

### 1500-m run

Time trials for the 1500-m run were performed at an indoor track. In the two trials, relative humidity (i.e., between 54 and 58%) and temperature (i.e., between 18 °C and 22 °C) were similar. Before the 1500-m run, a 5-min warm-up including stretching and/or light jogging was performed. Each participant ran the 1500 m alone to remove any feeling of competition. In the two trials, participants wore the same clothing and shoes and were instructed to complete the 1500-m run in the fastest time possible. The 1500-m run time was recorded by three experienced assessors using stopwatches. Assessors were instructed to start the stopwatch by a starting signal and to stop the stopwatch when the participant crossed the finish line with the body, excluding the head, neck, arms or legs. The median value from the three assessors was used as the time for the 1500-m run.

### Pulse wave velocity

We used automatic volume plethysmography (Form PWV/ABI; Fukuda-Colin Co., Tokyo, Japan) to measure baPWV. All measurements were taken after the participants had rested in the supine position for a minimum of 20 min, in reference to a previous report [25]. For each participant, electrodes for an electrocardiogram were placed on both wrists, a microphone was placed on the left edge of the sternum to detect heart sounds, and cuffs connected to a plethysmographic sensor and an oscillometric pressure sensor were wrapped around both arms and ankles to determine volume pulse forms and measure BP, respectively. We used a semiconductor pressure sensor to record the pulse volume waveforms. The sampling time was 10 s, with automatic gain analysis and quality adjustment.

We calculated the path length from the suprasternal notch to the measuring point in the brachial region (Lb) using the following equation: Lb = 0.2195 × height of the participant (cm) – 2.0734. To obtain the path length from the suprasternal notch to the ankle (La) from body surface measurements, we used the following equation: La = 0.8129 × height of the participant (cm) + 12.328. We calculated the distance between the two baPWV recording sites based on the height of the participant and anthropomorphic data for the Japanese population using the following equation:

$$ \mathrm{baPWV}=\left(\mathrm{La}-\mathrm{Lb}\right)/\mathrm{Tba}. $$

Two measurements were performed on each measurement day and the coefficient of variation for interobserver reproducibility of baPWV was 3%. In addition, the day-to-day reproducibility of measurements for PWV was 6%. These values mostly coincided with data from previous studies that identified the repeatability of PWV measurements [20–22, 25, 26].

### Resting brachial BP and HR

We measured resting HR and systolic/diastolic BP values simultaneously using electrocardiography and an automatic oscillometric device (Form PWV/ABI; Fukuda-Colin Co.), respectively. All data were recorded in triplicate while the participants were in the supine position. The pressure signal obtained by plethysmography was calibrated by equating systolic and diastolic BP values; this was then used to calculate mean arterial pressure [25].

### Statistical analysis

All data are expressed as mean ± SD. Statistical analyses were performed using Statistica software (SPSS ver. 24; SPSS, Chicago, IL). The assumption of a normal distribution was confirmed for all data using the Shapiro-Wilk test. Comparisons of these parameters were tested for significance using the paired *t-*test. Relationships between ΔV̇O_2_max, Δ1500-m run time (change from first to second visit) and ΔbaPWV were analyzed using Pearson’s correlation coefficients. The relative effect size for the performance data was calculated using Cohen’s *d* and defined as small (*d* = 0.2), medium (*d* = 0.5), or large (*d* = 0.8) [23]. In addition, 95% confidence intervals (CIs) are provided. Significance was set at the level of *P* < 0.05.

## Results

Ex1 revealed no significant differences in brachial BP or HR between higher and lower V̇O_2_max days (47.2 ± 4.6 mL/kg/min vs. 44.2 ± 4.7 mL/kg/min, *P* = 0.000002, *d* = 0.65, 95%CI = 45.3–49.1 mL/kg/min, Table 1). On the other hand, on days when participants had higher V̇O_2_max, mean baPWV was significantly lower (1031 ± 149 vs. 1076 ± 145 cm/s, *P* = 0.001, *d* = 0.75, 95%CI = 968–1094 cm/s, Table 1) than on days when V̇O_2_max was lower.

**Table 1:** Comparisons of V̇O_2_max, blood pressure and heart rate between days of higher and lower V̇O_2_max

	Higher V̇O_2_max day	Lower V̇O_2_max day
V̇O_2_max (mL/kg/min)	47.2 ± 4.6*	44.2 ± 4.7
baPWV (cm/sec)	1031 ± 149*	1076 ± 145
Systolic pressure (mmHg)	115±10	115±10
Mean pressure (mmHg)	83±8	82±7
Diastolic pressure (mmHg)	60±6	61±7
Pulse pressure (mmHg)	55±8	54±6
Heart rate (beats/min)	60±10	58±9

Values are means ± SD

* *P* < 0.01 vs. lower V̇O_2_max

*V̇O*_*2*_*max* maximal oxygen uptake, *baPWV* brachial-ankle pulse wave velocity

**Fig. 1:**
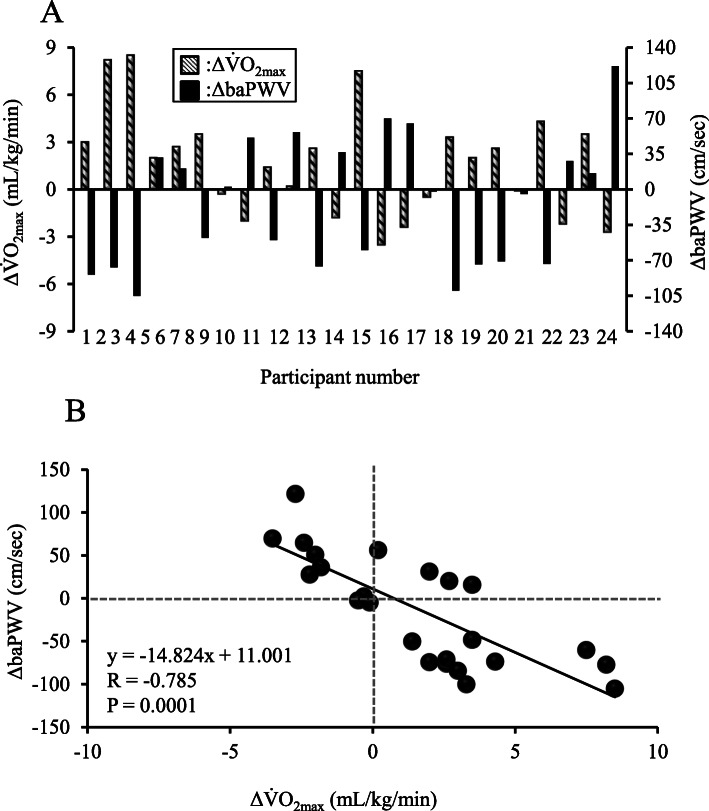
Individual changes in each Δ (change from first visit to second visit) baPWV and ΔV̇O_2_max between first and second visits (**a**) and relationship between ΔV̇O_2_max and ΔbaPWV from first to second visit (**b**). ΔV̇O_2_max: change in maximal oxygen uptake, ΔbaPWV: change in brachial-ankle pulse wave velocity

No significant differences in mean values were seen for V̇O_2_max, baPWV, brachial BP, or HR between first and second visits. However, individual variations in maximal aerobic capacity seems to be associated with those of arterial stiffness (Fig. 1a), and indeed a significant negative relationship was observed between individual changes in ΔV̇O_2_max and ΔbaPWV from first to second visit (r = − 0.785, *P* = 0.0001, Fig. 1b).

In Ex2, no significant differences were seen in brachial BP or HR between days of better and worse 1500-m run times (358 ± 40 s vs. 377 ± 51 s, *P* = 0.033, *d* = 1.12, 95%CI = 326–386 s; Table 2). On days when participants had a better 1500-m run time, mean baPWV was significantly lower (1003 ± 127 cm/s vs. 1068 ± 119 cm/s, *P* = 0.001, *d* = 1.83, 95%CI = 912–1094 cm/s, Table 2) than that on days when 1500-m run time was worse.

**Table 2:** Comparisons of 1500-m run time, blood pressure and heart rate between days of higher and lower 1500-m run times

	Higher time of 1500-m run day	lower time of 1500-m run day
Time of 1500-m run (sec)	358 ± 40*	377 ± 51
baPWV (cm/sec)	1003 ± 127*	1068 ± 119
Systolic pressure (mmHg)	113±10	114±11
Mean pressure (mmHg)	79±9	79±8
Diastolic pressure (mmHg)	63±9	63±8
Pulse pressure (mmHg)	50±6	51±4
Heart rate (beats/min)	64±6	64±5

Values are means ± SD

* P < 0.05 vs. lower time of 1500-m run day, *baPWV* brachial-ankle pulse wave velocity

**Fig. 2:**
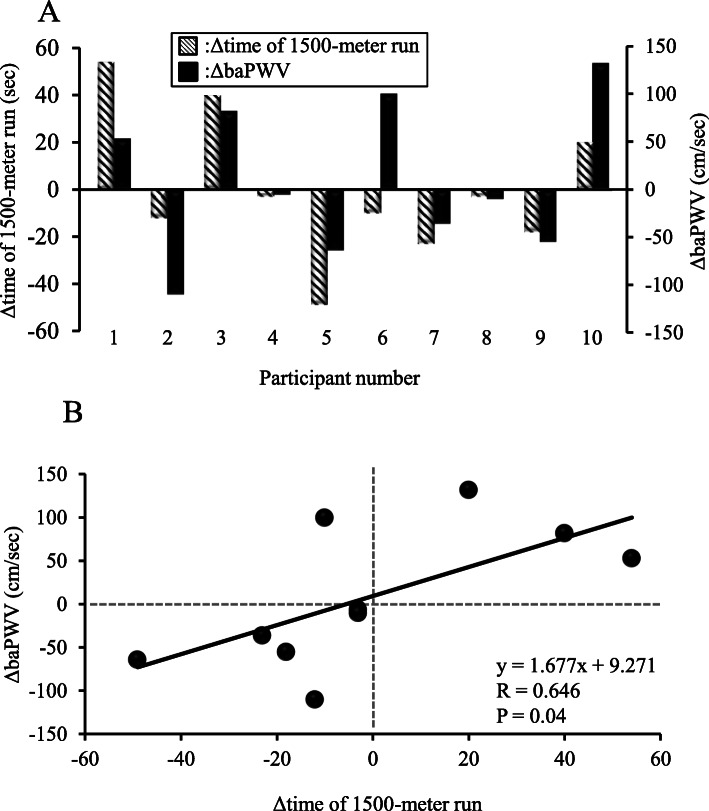
Individual changes in each ΔbaPWV and Δ1500-m run time between first and second visits (**a**) and relationship between Δ1500-m run time and ΔbaPWV from first to second visit (**b**). ΔbaPWV: change in brachial-ankle pulse wave velocity

No significant differences were seen in mean values for 1500-m run time, baPWV, brachial BP, or HR between first and second visits. However, as with the relationship between V̇O_2_max and PWV, individual day-to-day variations in maximal aerobic performance were similar to those of arterial stiffness (Fig. 2a), and thus a significant relationship was observed between individual changes in 1500-m run time and baPWV from first to second visits (r = 0.684, *P* = 0.029, Fig. 2b).

## Discussion

To the best of our knowledge, this is the first study to investigate the relationship between individual’s day-to-day variation in maximal aerobic capacity or performance and variation of arterial stiffness. The key novel results of this study were the identification of significant relationships between changes in V̇O_2_max or 1500-m run time and baPWV from first to second visits. These findings suggest that individual day-to-day variations in maximal aerobic performance are associated with variations in arterial stiffness.

Previous studies have already investigated whether individual cardiorespiratory fitness is associated with that of arterial stiffness [13, 17], however, these studies focused on a chronic determined relationship in a cross-sectional investigation. Therefore, the present study focused on an individual day-to-day variation and examined for the first time whether short-period variation in cardiorespiratory fitness is associated with that of arterial stiffness. In the present study, a significant negative correlation was identified between individual changes in V̇O_2_max or 1500-m run time and individual changes in baPWV from first to second visit (Figs. 1a, 2a). This finding suggests that individual day-to-day variation in maximal aerobic capacity or performance might be associated with variations in arterial stiffness. In other words, detectable alterations in PWV might offer effective indicators for predicting maximal aerobic capacity or performance. Our findings provide an important possibility for better understanding the role of fluctuations in arterial stiffness (i.e. vascular determinant) as a determinant of aerobic performance. Measurement of baPWV offers good reproducibility without needing a highly skilled technicians [27]. As a result, baPWV monitoring can provide an assessment of physical condition associated with sport performance for coaches and athletes in sports. Tomoto et al. [28] studied highly trained male collegiate endurance runners who participated in a training camp that involved relatively higher-intensity exercise and greater training volume. They found that systemic arterial stiffness was significantly increased after the camp. Monitoring training load in an athlete is generally considered important to determine whether the athlete is adapting well to the training program, as well as to minimize deteriorations in the physical condition of the athlete [29]. Given that changes in arterial stiffness were related to intense exercise training in the short-term, monitoring of day-to-day individual variation in PWV might be important for understanding the physical condition of an individual. Interestingly, increases or decreases in V̇O_2_max and 1500-m run time from first to second visit were not associated with variation of arterial stiffness in some participants (Figs. 1a, 2a). Further investigations may be necessary before PWV measurement can be routinely applied as an index of physical condition.

The mechanisms underlying the relationship between individual day-to-day variations in arterial stiffness and maximal aerobic capacity or performance remain unknown. However, various mechanisms should be considered in the background. Arterial stiffening causes excessive elevations in left ventricular afterload and increases myocardial work [30]. These cardiovascular adaptations to arterial stiffness decrease myocardial performance, and therefore exercise capacity, resulting in low cardiorespiratory fitness [19]. Even an acute decrease in arterial stiffness might thus contribute to improving maximal aerobic capacity. On the other hand, sympathetic modulation has been suggested to be beneficial as an index of conditioning in athletes [31–33]. The sympathetic nervous system is a key regulator of BP and HR, and abnormal activity in this system is related to increases in arterial stiffness [34, 35]. However, our study revealed no significant differences in resting BP or HR at the two visits separated by 1 month. In fact, no relationship has been demonstrated between sympathetic nervous activity and resting BP or HR among young healthy men and women [36, 37]. Variations in arterial stiffness due to sympathetic nervous system activity thus might plausibly influence maximal aerobic performance. Although these observations are not evidence of causal relationships, the results are interesting and provide scope for further investigation into the underlying mechanism.

V̇O_2_max is a major determinant of running performance and is highly correlated with running performance [38]. The relationship between individual variation in 1500-m run time, as an index of exercise performance, and that of baPWV was also investigated. Similar to V̇O_2_max, the present findings showed that individual variations in 1500-m run time were associated with variations in baPWV (Fig. 2). These findings further strengthen the evidence that individual day-to-day variations in maximal aerobic capacity and its associated aerobic performance are related to variations in arterial stiffness. The present findings provide important new physiological insights into variations in V̇O_2_max and subsequent exercise performance.

This study has several limitations. First, day-to-day variations in baPWV may include the repeatability of PWV measurement. The CV of day-to-day variation in baPWV was 6% in the present study. However, this value almost coincides with the results of previous studies that identified the repeatability of PWV measurement [20–22]. Moreover, all examinations were conducted in a standardized environment (i.e., measurement time, room temperature, etc.) to minimize measurement variability in this study. Thus, we believe that variation in baPWV represents that in arterial stiffness although a repeatability of PWV measurement. Second, this study examined the effect of differences in V̇O_2_max or 1500-m run time between only two visits over 1 month on baPWV. Therefore, no attempt was made to alter PWV to confirm actual effects of day-to-day variations in arterial stiffness on maximal aerobic capacity or performance due to individual variations in physical condition. Third, participants were recreationally active adults, but not athletes. Whether short-term variations in arterial stiffness are associated with exercise performance in athletes thus remains to be clarified. Finally, we were able to enroll only 10 healthy young adults (7 men, 3 women) into the Ex2 and included only 4 women in Ex1 and 3 women in Ex2. If there is a gender difference in the results, it is possible that this small ratio of woman subject modified our conclusion. However, a specific different result in woman subjects was not observed in the present study. In fact, one of 7 woman subjects has an opposite response (i.e. an increase in VO2 or exercise performance augments PWV) while three of 27 man subjects in the main finding. Thus, the ratio of subject who has an opposite response in women is the similar to that of men (14 and 11%, respectively). Therefore, an effect of the small ratio of woman subjects on our findings may be minimal but further investigations to confirm the effects of gender difference on the relationship between individual variations in maximal aerobic capacity or exercise performance and arterial stiffness are needed.

The present findings suggest that individual time-dependent variations in arterial stiffness are associated with changes in maximal aerobic performance. A single bout of exercise (e.g., self-myofascial release [39], stretching [26], whole-body vibration [40, 41] etc) reduces arterial stiffness. Reducing arterial stiffness using self-myofascial release even transiently, and stretching and/or whole-body vibration during warm-up might thus enhance aerobic performance. The present results potentially provide novel strategies to enhance exercise performance. Although carotid-femoral PWV is commonly considered the gold standard for the measurement of arterial stiffness, in the present study, arterial stiffness was assessed based on baPWV, which reflects changes in the stiffness of both the central (elastic) and peripheral (muscular) arteries. However, the baPWV technique offers good reproducibility, even without highly skilled technicians [27]. In addition, baPWV measures arterial stiffness in exposed limbs, and thus is not time-consuming. Given that changes in arterial stiffness were related to aerobic performance, baPWV might be predictive of body condition in the an individual, and may be applicable to conditioning for athletes.

## Conclusion

In conclusion, short-term variations in maximal aerobic capacity and performance may be related to variations in arterial stiffness. This finding indicates that individual day-to-day variations in the physiological condition associated with aerobic performance are related to variations in arterial vascular characteristics such as PWV. These findings indicate that PWV may offer a useful index for evaluating the physical condition of athletes.

## Supplementary Information

**Additional file 1.** Supplementary material. Short form of the International Physical Activity Questionnaire.

## Data Availability

The datasets used and/or analyzed during the current study are available from the corresponding author on reasonable request.

## Abbreviations

baPWV: brachial-ankle pulse wave velocity
BP: Blood pressure
CI: Confidence interval
CV: Coefficient of variation
HR: Heart rate
V̇O_2_max: Maximal oxygen uptake
